# Qatar's FIFA World Cup odyssey: A quest for legacy transforming a small nation into a global destination

**DOI:** 10.1016/j.heliyon.2024.e30282

**Published:** 2024-04-26

**Authors:** Mouna Hajjaj, Viktor Borodin, Diana Claudia Perțicas, Adrian Gheorghe Florea

**Affiliations:** a Faculty of Economics and Management, Hassan First University of Settat, Settat, Morocco; b Faculty of Mechanics and Mathematics, Taras Shevchenko National University of Kyiv, Kyiv, Ukraine; c Faculty of Economic Science, University of Oradea, Oradea, Romania

**Keywords:** Qatar, Destination image, Mega-events, Inbound tourism, Soft power, Content analysis

## Abstract

This study delves into the profound impact of mega-events on a destination's perception, focusing notably on Qatar's hosting experience during the FIFA World Cup 2022. Employing a qualitative longitudinal research approach, data is drawn from a variety of sources including Scopus articles, international studies, tourism records, newspapers, and interviews with diverse stakeholders such as visitors, tourism experts, executives, and government officials. Through an inductive content analysis of this extensive dataset, the authors identify key influencing factors. They meticulously examine a decade-long evolution using assessments from independent travel and tourism rating organizations alongside various metrics. The findings reveal a significant uptick in Qatar's allure and inbound tourism. Factors contributing to this surge are systematically categorized, with transportation and events infrastructure, hospitality and accommodation facilities, and media exposure emerging as consistent themes across host nations. Additionally, contextual factors like security/safety, culture/heritage, and diversity are highlighted for their pivotal role in shaping a destination's image. This research underscores how mega-events act as catalysts for reshaping Qatar's destination identity and attracting inbound tourism, as evidenced by a range of indicators contingent upon maintaining both universal and contextual factors. It emphasizes the importance of a strategic vision that nurtures these factors over time, aligning with the destination's life cycle to forge a lasting legacy. Furthermore, the study illuminates how mega-events can bolster soft power for small countries lacking significant natural or historical attractions, providing valuable insights for policymakers and marketers to devise effective strategies.

## 1 Introduction

In recent decades, a notable trend has emerged in destination management, driven by the imperative to avoid stagnation and decline [1]. Destinations worldwide are actively pursuing new events and bidding for existing ones to inject vitality into their tourism offerings [2,3]. Central to this dynamic is the concept of destination image —a multifaceted construct shaped by a myriad of influences, including the impact of mega-events theorized as *changed induced image* and the resulting human experiences [4]. Among these events, sports events reign supreme in visibility and impact due to their massive scale and extensive audience reach [5]. They present nations with a rare opportunity to rebrand themselves on a global stage, mitigating information asymmetry and reshaping international perceptions. Media coverage plays a pivotal role in this transformative process, evolving through three distinct phases: (1) the pre-event stage, characterized by initial perceptions, often emphasizing negative aspects; (2) during the event itself, when visitation peaks; and (3) the post-event phase, marked by strategic efforts to sustain the destination's appeal. Foreign media tend to spotlight negative aspects, considering them newsworthy, while local media typically convey a more positive narrative [6]. The varying influence of media sources, with reputable foreign channels wielding substantial power due to their broad reach and established reputation, compared to local counterparts, underscores the complexity of perception formation [7]. This interplay is further reflected in the cultivation theory, where limited information and firsthand experiences shape perceptions [7].

However, scholarly opinions diverge on the extent to which mega-sport events influence destination image, with some even questioning their tangible impact [8]. This research seeks to contribute clarity to the scholarly discourse on the effects of mega-events on destination image. Specifically focusing on Qatar's FIFA World Cup (FWC) hosting experience, this study aims to examine the multifaceted impacts of mega-events on destination image and tourism development. Qatar's selection as the focal point of this research is underpinned by several compelling factors, rendering it an ideal case study for exploring the nexus of mega-events, destination image, and tourism development. Despite its relatively small population and land area [9], Qatar has distinguished itself as one of the world's wealthiest economies, boasting a consistently high GDP per capita. The decision to host such a prestigious event reflects Qatar's ambitious agenda to diversify its economy away from reliance on oil revenues towards becoming a premier tourism destination, aligning with the strategic objectives outlined in the National Vision of Qatar (QNV) 2030 [10]. The substantial investment exceeding $200 billion in hosting the FWC 2022 underscores Qatar's unwavering commitment to the event and its broader strategic goals. The involvement of multiple cities across Qatar in hosting the games underscores the nation's capability to mobilize resources and infrastructure at a national level, offering visitors a diverse and appealing tourism experience. Furthermore, Qatar's track record in hosting mega-events and its ongoing efforts in global sports diplomacy underscore its ambition to leverage sports as a tool for soft power projection [11]. Qatar has successfully hosted mega-events and continues to play a central role in global sports diplomacy, evident through events such as the annual *Doha GOALS* conference and the *Securing Sport* symposium organized by the International Centre for Sport Security (ICSS). Beyond hosting events, sport stands out as Qatar's primary avenue for global engagement, exemplified by its ownership of prominent football clubs like *Paris Saint-Germain* and its active sports sponsorship initiatives through entities like *beIN Sports*, a global sports broadcasting network. These strategic endeavors position Qatar as a global sports and tourist powerhouse.

This study seeks to address the existing gap in understanding the long-term effects of mega-events on destination image, particularly in the context of small countries leveraging soft power to exert disproportionate global influence. Despite the growing significance of mega-events in shaping destination perceptions, existing research falls short in providing a comprehensive exploration of this topic. Moreover, Qatar, as a relatively understudied tourism destination, offers a unique opportunity to unravel the intricacies of destination image following the hosting of mega events.

By delving into Qatar's experiences and strategies in hosting mega-events, this study aims to illuminate the evolving dynamics of destination management and the pursuit of soft power objectives. Specifically, by examining media coverage patterns across pre-, during, and post-event phases, the study seeks to understand Qatar's *organic image* highlighting and deliberate efforts to enhance its *induced image*. Moreover, considering the phenomenon of the *modified induced image*, the research will explore how firsthand experiences of Qatar influence destination image, addressing a significant gap in the literature regarding the long-term impacts of mega-events on destination perceptions, particularly in the context of smaller nations.

Given the significance of Qatar's hosting of the FWC 2022, this study offers a unique opportunity to unravel the complexities of destination management and soft power projection in the contemporary landscape. By contributing to a deeper understanding of the interplay between mega-events, small countries, and soft power, this research aims to enrich the existing literature in the field of destination management and provide valuable insights for policymakers, practitioners, and scholars alike.

## 2 Literature review

The conceptual framework of this study draws upon various models and frameworks from the realms of marketing, brand management, destination identity, and tourism studies. These frameworks serves as theoretical underpinnings for comprehending the intricate dynamics involved in the impact of mega-events on destination image and inbound tourism. One such framework is the co-branding theory, commonly utilized in marketing and brand management. Co-branding entails a strategic alliance between distinct entities to create a unified and mutually beneficial brand identity, leveraging shared values, aims, or market segments [11]. This framework sheds light on how mega-events like the FWC can be harnessed to bolster the host country's image by transferring the event's positive associations to the host country in a complex pairing. Xing and Chalip [12] assert that the success of co-branding hinges on meticulous alignment of schemas, representing mental frameworks that aid individuals in interpreting objects or experiences. Notably, the FWC and Qatar have emphasized diversity by celebrating teams and fans from diverse backgrounds, fostering a sense of unity on a global scale. Qatar's substantial investments in cutting-edge safety measures and health standards have further contributed to creating a safe and enjoyable experience for all attendees. Incorporating Qatari culture and heritage —traditional art, music, and hospitality—into the competition has added a unique and authentic dimension to the event, making it a celebration of diversity, safety, and cultural legacy. This shared paradigm strengthens the resonance of the event and enhances Qatar's global image. McDaniel [13] suggests that a shared schema can also facilitate the transfer non-common properties between brands, in the light of the *match-up effects*, emphasizing the importance of alignment in avoiding dissonance.

Moreover, discussions on strategic destination identity management draw insights from Kepferer's concept of *sender-side* identity management [14] and the *Olympic effect* identified by Rose and Spiegel [15]. These frameworks underscore the long-lasting impact of mega-events on a destination's identity and its potential to convey positive signals to global audiences. For instance, Rose and Spiegel [15] discovered that the act of bidding to host a mega-sport event, rather than hosting it, resulted in increased exports, highlighting the lasting effects of mega-events on a destination's identity. Destination image, from the receiver's perspective, is a multifaceted construct shaped by visitors' emotions and impressions, Transcending managerial control. It manifests itself across three distinct dimensions [4]: (1) the *organic image* influenced by independent media coverage and word-of-mouth, (2) the *induced image* meticulously crafted through promotional efforts, (3) and the *modified-induced image* shaped by personal experiences on-site, particularly during mega-events.

To further refine the conceptual framework, existing models and frameworks are considered, including Gartner's Destination Image Formation Model [16]. This model suggests that destination image is shaped by cognitive, affective, and conative processes. The *cognitive dimension* encompasses the accumulation of knowledge and information about a destination, influenced by factors such as media coverage and word-of-mouth. The *affective dimension* pertains to emotional responses and perceptions towards the destination, shaped by personal experiences and promotional efforts, aligning with *pull-push* theory [17,18]. According to this theory, individuals are either *pulled* towards a destination by attractive features, or *pushed* to travel due to factors in their home environment [18]. The Psychological Continuum Model (PCM) [19] further elucidates the formation of attitudes along a chronological progression, emphasizing the interplay between external stimuli (*pull factors)* and internal motivations (*push factors)*. Finally, the *conative dimension* concerns behavioral intentions and decisions regarding travel, influenced by both pull and push factors. The initial stage of this theory, known as the *pre-visitation phase, encompasses* the awareness and the attraction stage of the PCM [19]. Initially, awareness regarding a destination increases, as potential visitors considerate traveling there, influenced by internal factors shaped by pull factors. Subsequently, the attraction stage ensues, significantly influencing travel decisions as visitors consider external push factors. Transitioning into the *during visitation phase*, we encounter the development of the attachment stage. Here, actual visitors immerse themselves in their experience, deriving enjoyment and forming a positive evaluation. This stage sets the groundwork for the *post-visitation phase,* characterized by allegiance—an enduring sentiment encapsulating intentions to revisit.

Building upon these theoretical foundations, the conceptual framework integrates diverse components to offer a comprehensive understanding of the influence of mega-events on destination image and inbound tourism. It identifies universal pathway factors such as transportation and events infrastructures, accommodation and hospitality facilities, and media exposure as critical elements shaping perception of mega-events and their impact on destination image. Additionally, contextual factors varying across countries, including security/safety, culture/heritage, and diversity, are recognized as significant determinants amplifying or mitigating the overall influence of mega-events on destination image and inbound tourism. The relationships within the framework are dynamic and multifaceted with transportation and events infrastructure facilitating event hosting, accommodation and hospitality facilities enriching visitor experiences, media exposure amplifying global visibility and contextual factors influencing visitors’ perceptions and future travel intentions. By considering of the interplay between these factors, the framework offers valuable insights for event organizers, destination managers, and policymakers striving to leverage mega-events for tourism development and promotion.

The examination of relationships within the defined factors illuminates potential dynamics within the conceptual framework. Investment in transportation and events infrastructures are anticipated to enhance destination hosting capacity for mega-events, leading to increased accessibility and visitor inflow. Quality accommodation and hospitality facilities are expected to elevate visitor satisfaction and contribute positively to destination image. Moreover, heightened media exposure during mega-events is poised to bolster destination visibility and awareness globally, fostering favorable perceptions among potential tourists. Contextual factors such as security/safety, culture/heritage, and diversity are also anticipated to play pivotal roles, shaping tourists' decision-making processes and overall satisfaction levels. These intricate relationships offer valuable insights into the complex interplay between various factors shaping the influence of mega-events on destination image and inbound tourism.

## 3 Method

### 3.1 Research design and data collection

This study employs a qualitative longitudinal method to analyze topic-related materials. The researchers opted to extend data collection from the announcement of the World Cup 2022 hosting country in December 2010, until one year after the conclusion of the games in December 2023. This pre-during-post comparison captures the event's multifaceted impact to provide comprehensive insights.

**Fig. 1:**
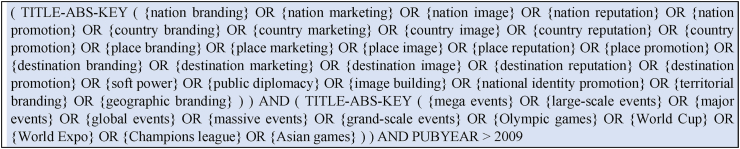
Query performed on Scopus.

To ensure the scientific rigor of the collected articles and meet specific criteria, the researchers selected papers published in peer-reviewed journals indexed in Scopus database. A comprehensive search query tailored to the study's scope and the selected time period was used to query the Scopus database. The query included key terms such as 'nation branding,' 'country image,' and 'place marketing,' to encompass literature on strategic positioning of nations or places globally. Additionally, phrases like 'mega events,' 'Olympic Games,' and 'World Cup' were included to retrieve studies on the relationship between hosting mega-events and its impact on nation or place branding. This inquiry aims to uncover scholarly insights into how prestigious events reverberate across national or place identities, images, and promotional efforts. The query yielded 291 papers. Fig. 1 above is the exact query performed on Scopus.

The inclusion of the Scopus query in Fig. 1 visually illustrates the search strategy used in this study, enhancing transparency and facilitating replication by providing a clear and reproducible method for future research. Moreover, it aids reader comprehension by offering a concise overview of the literature review process within the broader research framework.

Furthermore, various reports and indices providing insights into global travel trends, country reputation and competitiveness was considered, where available. Table 1 provides a comprehensive overview of the reports and indices considered spanning the years 2013–2023 and encompassing 63 documents from diverse sources. These sources include renowned institutions such as Anholt-Ipsos, Bloom Consulting, Brand Finance, Future Brand, Institute for Economics & Peace, Kearney, U.S. News, World Travel & Tourism Council, and World Economic Forum. Each source is accompanied by the title of the report or index it provided, along with the years covered. For example, the Anholt-Ipsos Nation Brand Index covered the years 2020–2023, while the Bloom Consulting Country Brand Ranking Tourism Edition covered the years 2013–2020 and 2022 to 2023. This collection of documents offers a comprehensive view of the global travel trends, country reputation, and competitiveness, facilitating in-depth analysis and informed decision-making in these domains.

**Table 1:** Reports and indices considered for global travel trends, country reputation, and competitiveness.

Source	Title	Years
Anholt-Ipsos	Nation Brand Index	2020–2023
Bloom Consulting	Country Brand Ranking Tourism Edition	2013–2020; 2022–2023
Brand Finance	Global Soft Power Index	2020–2023
Future Brand	Country Index	2019–2020
Institute for Economics & Peace	Global Peace Index	2013–2023
Kearney	Global Cities Report	2014–2022
U.S. News	Best Countries in the World	2017–2023
World Travel & Tourism Council	Trending in Travel	2022
World Travel & Tourism Council	Cities Impact	2019; 2022
World Travel & Tourism Council	Economic Impact	2019; 2022
World Economic Forum	Travel & Tourism Development Index	2013–2021

Moreover, Table 2 presents a comprehensive compilation of reliable information sourced from official channels, enriching the dataset with valuable insights and data directly from the Qatari government, as well as from FIFA and reputable news sources. The data covers multiple years, offering a detailed understanding of various aspects related to Qatar's development, particularly in the context of hosting the FWC 2022. The sources include the General Secretariat for Development Planning, providing insights into Qatar's long-term vision through the Qatar National Vision 2030. Additionally, the Qatar Development Bank offers detailed information on the tourism sector in Qatar. The Tourism Sector Authority contributes with the Qatar National Tourism Sector Strategy 2030, outlining plans for the country's tourism development. Furthermore, governmental entities such as the Ministry of Communications and Information Technology and the Ministry of Transport provide valuable information on infrastructure and connectivity initiatives, including the Connected Tournament and the Transportation Master Plan for Qatar (TMPQ). Additionally, FIFA's reports, including the Bid Evaluation Report and the Final Sustainability Report, offer crucial insights into Qatar's preparations and execution of the FWC 2022.

**Table 2:** Official Data and Insights sources.

Source	Title/Content	Years
General Secretariat For Development Planning	Qatar National Vision 2030	2008
Qatar Development Bank	Tourism sector in Qatar	2021
Tourism Sector Authority	Qatar National Tourism Sector Strategy 2030	2021
Ministry of Communications and Information Technology	Connected Tournament	2023
Ministry of Transport	Transportation Master Plan for Qatar (TMPQ)	2006
FIFA	Bid Evaluation Report: Qatar	
FIFA	FIFA WC Qatar 2022 at a Glance	2023
FIFA	Final Sustainability Report	2023
Al Jazeera	News report about the FIFA WC 2022	2010–2023
BeIN Sports		2010–2023
CNN		2010–2023
BBC		2010–2023
The New York Times	Articles about the FIFA WC 2022	2010–2023
The Guardian		2010–2023

Furthermore, meticulous data triangulation is employed to ensure a comprehensive and nuanced understanding by integrating multiple sources of data. By incorporating both primary and secondary sources, the study endeavors to mitigate the limitations inherent in relying solely on a single data stream. The researchers seek to enhance the reliability and validity of the findings by cross-validating information obtained from different perspectives. This approach fosters the credibility of the research outcomes and provides a nuanced interpretation of the findings grounded in the context of Qatar's hosting of the World Cup.

The researchers conducted interviews with visitors to the 2022 World Cup or upcoming events, as well as stakeholders involved in mega-event organization, tourism industry experts, and government officials in Qatar. Snowball sampling was employed to identify participants, starting with individuals known to the researchers. These initial contacts were then asked to recommend other experts, officials, and visitors with valuable insights into Qatar's tourism landscape and destination management. A total of 7 interviewees participated, offering diverse perspectives on Qatar's tourism landscape and the implications of hosting the 2022 FWC. Each of the three distinct categories of visitors –sports fans, travel influencers, and corporate travelers– was represented by three individuals, providing unique insights derived from their varied backgrounds and interests. Additionally, one seasoned hospitality executive resort chains shared his expertise on executive-level decision-making and operational strategies. One tourism expert with consultancy experience enriched our understanding of destination branding and event management strategies. Moreover, one government official from the Ministry of Culture and Sport and one other from Qatar Tourism, a government body under the umbrella of the Prime Minister's Office, participated in the interviews. They offered insights into governmental roles and policies related to cultural and historical attractions, as well as business and MICE tourism. The participants hailed from diverse nationalities, underscoring the global appeal of Qatar as a tourist and business destination. Their ages ranged from 28 to 45 years, with an average age of 36, ensuring a broad spectrum of perspectives. Interviews were transcribed, translated, and coded for analysis, allowing for a thorough examination of the collected data. The data collection process involved conducting one-on-one, in-depth interviews following the collection of secondary data.

### 3.2 Data analysis

The researchers defined clear research questions to maintain focus throughout the analytical process, with frequent revisits to match queries with evolving insights. Inter-coder discussions and a common coding manual create a unified understanding and interpretation of the data, ensuring the integrity of the results.

This study adopts inductive content analysis method. The researchers read the data, inductively label key phrases, and categorize codes. This iterative process helped refining and reviewing the factors retrieved from the data. This approach guaranteed the reliability and validity of the findings. The analysis was performed in NVivo software, which helps with reading, retrieval, and data management for developing the conceptual model of this research.

## 4 Results

**Fig. 2:**
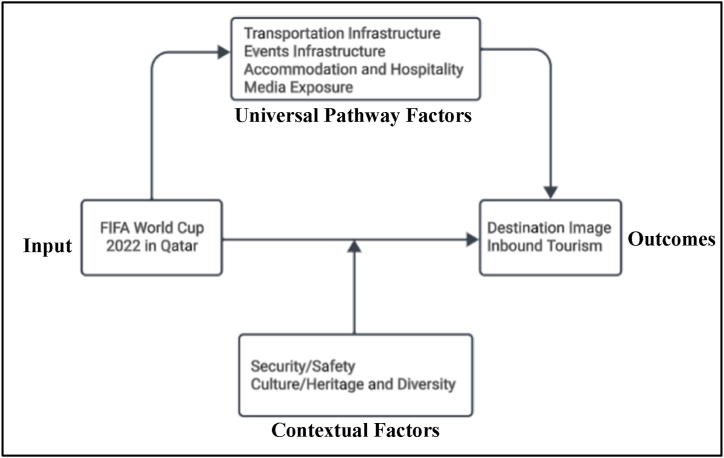
Conceptual framework of the research model.

The results of this study underscores the significant influence of the Qatar World Cup in shaping destination image and increasing inbound tourism. As illustrated in Fig. 2, the study highlights universal pathway factors such as transportation and events infrastructures, accommodation and hospitality facilities, and media exposure as key influencers. Additionally, contextual factors varying across countries such as security/safety, culture/heritage, and diversity significantly affect the overall influence.

### 4.1 Universal pathway factors

#### 4.1.1 Transportation infrastructure

Qatar's robust transportation infrastructure, recognized internationally, positions the country as a vital air transportation hub, ranking 23rd internationally in the World Economic Forum's 2023 Travel & Tourism Competitiveness Index [20]. This recognition acknowledges Qatar's commitment to maintaining a strong and efficient air transportation system, essential for the success of mega-events like the World Cup. In the same year, US News and World Report noted Qatar's capital accessibility (58.7) and well-developed infrastructure (68.8) [21]. Qatar's forward-thinking strategy helped build a complete support system for the World Cup and its aftermath, ensuring mega-event success and creating a lasting legacy that enhances the nation's appeal to visitors and travelers worldwide. Qatar's commitment to a strong and efficient transportation system is shown by Doha airport's capacity growth from 30 to 50 million passengers per year.

During the tournament, free public transportation was provided for *Hayya* Card holders and workforce accreditations, backed by social media and text messages (SMS) for travel guidance, including infographics and maps. FIFA in its final sustainability report [22] revealed that a fleet of 3,000 buses, including 900 electric ones, efficiently transported 5 million spectators facilitated by shuttle services operating every 5 and 15 min during peak times. Additionally, new rail and metro systems connect all three host cities, which is noteworthy. Doha Metro, with 104 trains and increased frequency recorded 17 million journeys [22] – a 300 % increase with an average of 600,000 passengers per day. Operating up to 21 h daily, it catered to the needs of spectators, workforce, and volunteers. Micro-mobility options such as e-scooters, e-bikes, and conventional bicycles were available for hire across Qatar, excluding pedestrianized areas to ensure safety. Taxis and rideshare services were enhanced, focusing on high-demand areas like airport pick-up. With expanded capacity, 795,000 spectators opted for ridesharing [22]. The success of Qatar's transportation infrastructure was evident in the sentiment expressed by a sports enthusiast from the study: "*Even though there were tons of people for the matches, the transportation system handled the crowds surprisingly well*." This firsthand observation underscores the meticulous planning and execution demonstrated by Qatar in managing large-scale events and ensuring a seamless travel experience for attendees. On the other hand, to meet the increased demand, Doha International Airport, along with *Hamad* International Airport, temporarily opened for commercial flights, handling 32,000 arrivals and departures daily [22]. Those initiatives serve a dual purpose: helping the World Cup run smoothly and improving the region's transportation network as a lasting impact.

#### 4.1.2 Events infrastructure

Qatar has risen as a regional frontrunner in holding a diverse range of events, spanning corporate and leisure sectors, conferences, thematic fairs, festivals, and, notably, sporting competitions. Beyond the FWC, Qatar has showcased its event hosting prowess by organizing the Swimming World Cup (2014), Handball World Cup (2015), Road Cycling World Cup (2016), Gymnastics World Cup (2018), Arab Cup (2021) and the AFC Asian Cup (2023). Annually, Qatar participated in esteemed international circuits such as World Tennis, Golf, and MotoGP.

The country's dynamic engagement is evident in advertisement of 849 international events scheduled for 2023 on the website “iloveqatar.net” [23] alongside 112 upcoming events published on Qatar National Tourism Council website “visitqatar.qa” in November [24]. Qatar's strategic investment in sports and mega-events has proven fruitful, particularly for the national tourism industry. This shift away from traditional business trips and a lack of natural attractions highlights Qatar's proactive approach to broadening its appeal beyond corporate travel.

Furthermore, Qatar's ranking of 32nd in ICT in the World Economic Forum's 2023 Travel & Tourism Competitiveness Index [20] underscores its commitment to technological infrastructure. Similarly, in the same year, US News and World Report praised Qatar's digital infrastructure, scored 61,3 out of 100 [21]. These measures reflect Qatar's readiness to embrace the digital age and leverage technology to enhance event experiences, solidifying its status as a premier global events destination.

#### 4.1.3 Accommodation and hospitality

Qatar's cruise sector stands as a cornerstone of the country's long-term tourism ambitions [25], with significant strides made to facilitate its growth. Notably, the extensive renovation of Doha Port enables it to accommodate two superships concurrently, welcoming up to 12,000 cruise guests daily [26].

Ranking 5th globally in customer orientation in the Travel & Tourism Competitiveness Index [20], Qatar's hospitality prowess is underscored, highlighting its dedication to providing a warm and welcoming experience for tourists. The nation's commitment to enhancing tourist facilities, including hotels and restaurants, is evident in its diverse initiatives aimed at creating an enticing tourism landscape. Qatar jas constructed 240 4- and 5-star hotels to improve its lodging infrastructure [22], emphasizing its efforts to elevate the quality and standards of hospitality offerings.

As the tourism sector flourishes, Qatar strategically positions itself as a business hub, as affirmed by a corporate traveler in the interview: "*The conference facilities in Doha are top-notch (…) Qatar is definitely positioning itself as a business hub*." This focus on providing world-class conference facilities aligns with Qatar's broader vision of diversifying its economy and attracting both business and leisure travelers.

The World Travel & Tourism Council's "Cities Impact 2022" report highlights Doha's significant contribution to Qatar's GDP, with the travel and tourism sector accounting for 90 % in 2019 and 91 % in 2021 [27]. By 2024, the sector's GDP is projected to return to 2019 levels, with annual growth expected to surpass the regional economy's growth rate between 2022 and 2032.

During the FWC Qatar 2022, the Supreme Committee for Delivery & Legacy collaborated with an accommodation agency to streamline housing options on a dedicated portal, offering ticketholders a *one-stop-shop* for browsing and booking. Innovative temporary accommodation solutions, including hotels, apartments, villas, holiday homes, fan villages, and floating hotels, were identified to complement permanent accommodation options. With peak occupancy reaching 90 % [22], rooms were consistently available, with the FIFA Hotel Agreement securing almost 84,000 rooms exceeding the 60,000-room requirement [22].

#### 4.1.4 Media exposure

Media coverage of Qatar's World Cup hosting is multi-faceted, reflecting different interpretations across various outlets, a phenomenon termed as ‘*competing frames’* by Chong and Druckman [28]. A comparison between local media in Qatar (Al Jazeera, BeIN Sports), and global news organization (CNN, BBC, The New York Times, and The Guardian), from winning the 2022 World Cup bid to the beginning of the games reveals a contrast in framing, with local media presenting a positive view while global media offer more critical perspectives. On the other hand, the cultivation theory [31, p: 7] addresses the cumulative, long-term effects of media exposure.

Jones [29] conducted a study on UK headlines during this period, finding that Qatar appeared 1,735 times, with World Cup articles dominated 40 % (685) of the coverage. These articles significantly shape Qatar's reputation in the British press, supporting the cultivation theory. Jones also identified recurring themes in the coverage, with human and labor rights, sports, 2017 Gulf Crisis, corruption and bribery, and aviation being the most prominent. The majority of British media coverage was unfavorable, with 66 % (454) critical, 29 % (201) neutral, and 5 % (33) positive. Negative articles predominantly addressed workers' rights (36 %), and corruption and bribery (25 %), with some mentioning losing the World Cup (9 %), or highlighting LGBTQ + rights (4 %).

The media effect on World Cup attendance in Qatar is significant. In a survey of 481 Swedish fan association members conducted by Andersson, Bengtsson, and Svensson [30], 62.8 % of respondents stated that the media influenced the decision to attend the World Cup in Qatar. Qatar's worldwide image and trustworthiness have suffered from Western media coverage of its host country status. Brannagan and Giulianotti [31] predicted that this negative portrayal could contribute to a period of soft disempowerment for Qatar. However, despite these challenges, the researchers suggested that such a situation could also serve as an opportunity for the state to respond positively and rebuild its soft power capabilities over time.

Despite these challenges, Qatar's attempts to combat negative media portrayals and enhance its global image have been extensive. As expressed by the Qatar Tourism representative during the interview, "*The negative media narrative was definitely a challenge in the lead-up to the World Cup (…) We worked hard to counter these narratives by promoting authentic stories and experiences from Qatar.*" Qatar has heavily pushed its tourism business to alter media-driven World Cup attendance perceptions. Qatar has utilized social media platforms and collaborated with major media outlets on campaigns such as the *Qatar, Qurated for You* initiative in 2018 [25]. This strategy aims to present a diverse and positive perspective of Qatar to a global audience. In addition to digital advertising, Qatar has employed traditional print media, with ads appearing in 10 countries and 14 airports showing its dedication and proactivity by reaching 250 million people [25]. As aptly summarized by the same participant, "*We welcomed open dialogue and constructive criticism, but we also challenged unfair portrayals and misinformation. We believe the World Cup ultimately helped to change the perception of Qatar for many people*."

### 4.2 Contextual factors

#### 4.2.1 Culture/heritage and diversity

Qatar's cultural heritage and diversity significantly contribute to its global image. Ranking 37th in the Travel & tourism Competitiveness Index [20], Qatar excels in its capacity to attract foreign labor (4th) and discover high-quality human resources domestically and internationally (7th), showcasing its worldwide appeal and connectivity. Qatar's *‘soft power’* scored 49,9 out of 100 in the 2023 Brand Finance report, placing it 24th internationally [32]. Despite this, Qatar faces challenges in heritage preservation, ranking 67th in ‘*heritage*’, in the same report. Low scores in criteria such as *‘superb food’* (5.8), *‘many cultural attractions’* (19.1), and *‘geographic attractions’* (9.6) contribute to this ranking. US News and World Report awarded the country a score of 17.8 out of 100 based on the criteria of being *‘culturally significant in terms of entertainment’* [21]. However, Qatar compensates with high scores in criteria like *‘modern’* (77.8), *‘prestigious’* (66.3) attributes, and a perfect *‘trendy’* score (100), placing 21st in *‘*Cultural Influence*’* [21]. Efforts to develop UNESCO Heritage Sites, like *Al Zubarah* and *Ras Brouq*, underscore Qatar's commitment to preserving its rich history [33,25]. Local attractions like *Souq Waqif* and the *Katara Cultural Tourism Hub* further enhances Qatar's appeal as a diverse destination [25].

Qatar is ranked 2nd globally in *‘movers’*, with high scores for attributes like *‘different’* (100), *‘distinctive’* (86.5) and *‘unique’* (91.8), and 22nd on *‘agility’* thanks to its favorable criteria *‘dynamic’* (79.7) and *‘progressive’* (60.8). Qatar's distinct identity appeals to travelers seeking unique experiences. Investments in cultural and recreational activities, contemporary architecture, cultural institutions, and a growing hotel business aim to attract tourists seeking a blend of tradition and modernity. This aligns with the growing trend among travelers where 40 % want to explore new destinations [34] and 69 % of travelers desire to see lesser-known destinations [35].

The FWC 2022 showcased Qatar's openness, and genuine hospitality. Lionel Messi's image carrying the trophy adorned in the *bisht* symbolized Qatar's embrace of the world, fostering global connections and reconciling Arab and Western cultures. This gesture emphasized Qatar's commitment to showcasing its culture while embracing the global community. Industry expert stress the importance of "*Balancing cultural preservation and tourism development will be important for Qatar's sustainable growth*". emphasizing the significance of preserving cultural heritage while fostering tourism development for Qatar's long-term sustainability. Aligning with this sentiment, a Ministry of Culture and Sports decision maker affirms that "*efforts are focused on showcasing Qatar's rich heritage alongside [the]modern advancements,*" indicating a concerted effort to harmonize tradition with progress to showcase Qatar's unique identity to the world.

#### 4.2.2 Safety/security

Horn and Breetzke [36] emphasize the crucial role of safety management in destination promotion, a principle that aligns with Qatar's unwavering commitment to security. Qatar's reputation for safety is exceptional, with the WEF naming it the second-safest nation in 2015, just behind Finland [37]. Despite facing challenges such as the 2017 blockade, Qatar has demonstrated resilience, consistently ranking in the top 10 for safety and security. In 2021, views of Qatari leaders and security improved significantly [38]. By 2023, Qatar has ascended to the 27th position in international rankings for *‘quality oef life’* according to the US News and World Report criteria [21]. This rise was propelled by high scores in *‘safety’* (53.5) and *‘political stability’* (43.8). A sentiment echoed by a travel influencer in the interview reflects the widespread perception of safety in Qatar, stating, "*I felt safer in Qatar than I do in my own city back home. There was a visible police presence but not overwhelming. They seem more focused on keeping things smooth and helping people if needed (…) I never felt like I had to worry about stuff, even when I was out at night with all my camera gear.*"

The 2023 Global Peace Index further underscores Qatar's commitment to peace, with the country consistently being the most peaceful country in the area and ranking among the top 25 most peaceful nations globally since 2008 [39]. Qatar's rise from 30th in 2019 to 21st in 2023 [39] is attributed to improved political relations with neighboring countries since 2021. Peace in Qatar stems from factors such as political stability, international conflict resolution, and UN peacekeeping funding.

Key indicators evaluated by the Global Peace Index, such as *‘perceived criminality in society’* and *‘security officers and police’, have remained* unchanged over the past decade. Qatar's low score of 1.343 for *‘perceived criminality in society’* indicates a minimal level of criminal activity as analyzed by Economist Intelligence Unit analysts. Additionally, the score of 3.304 of *‘security officers and police’* as as by the UN Survey of Crime Trends and Operations of Criminal Justice Systems, reflects Qatar's dedication to maintaining a high level of the criminal justice system. Consequently, Qatar ranks 9th globally in the *‘Societal Safety and Security’* domain for 2023.

### 4.3 Outcomes

#### 4.3.1 Destination image

Qatar's destination image is evaluated through various metrics, providing insights into its global attractiveness. Ranked as the 24th most popular destination on TripAdvisor [40], Qatar demonstrates its widespread appeal, particularly as the 4th most popular destination in the Middle East [40]. The Global Attractiveness Index further illustrates Qatar's evolving image, rankinganks 23rd with a score of 63.1 out of 100 in 2023, a positive trend from previous years [41]. In addition, Qatar rose one spot to 41st in the World Economic Forum's Travel & Tourism Competitiveness Index [20].

Despite these positive rankings, Qatar's *‘familiarity’* dropped from 46th to 59th in Brand Finance's 2021 assessment [38], possibly due to the FWC's November–December postponement [41], which raised concerns about harsh heat and alcohol restrictions [41]. However, strategic planning has improved *‘familiarity’*, *‘reputation’*, and *‘influence’* key performance indicators (KPIs), leading to Qatar's inclusion in the top 25 for the first time [32]. Reflecting on the FWC, a participant from the Ministry of Culture and Sports articulated, "*The World Cup was a triumph, not only in terms of sporting competition but also in fostering cultural exchange and mutual understanding. We aim to capitalize on this momentum to cultivate a more positive image of Qatar in the years ahead.*" This sentiment underscores Qatar's commitment to leveraging mega-events like the FWC to enhance its global reputation and foster cultural exchange [42].

Qatar ranks 18th in Asia in the Bloom Consulting Country Brand Ranking Tourism Edition 2022-23 [43]. Qatar ranks 26th overall in 2023, according to US News and World Report [21]. However, Qatar ranks low for *‘adventure’* due to its geographical characteristics lacking diverse terrains for activities like hiking and mountaineering [21]. The flat geography and lack of elevated landscapes also hinder its appeal to photographers and influencers seeking panoramic vistas and distinct ecosystems, Qatar is not considered as *‘scenic’* (10.9) [21]. Additionally, Qatar's weather, with a score of 5.0 for *‘nice temperature’* may not be ideal for adventure seekers due to excessive heat.

In terms of global perception, Qatar improved its position in Future Brand's 2021 evaluation after addressing unfavorable attention regarding its handling of migrant workers, effectively using the FWC to change worldwide attitudes [44,45]. The Anholt-Ipsus score also reflects Qatar's improving global image, ranking 50th in 2021, 53rd in 2022 and regaining a place in the top 50 with a score of 55.24 in 2023 [46,47].

**Fig. 3:**
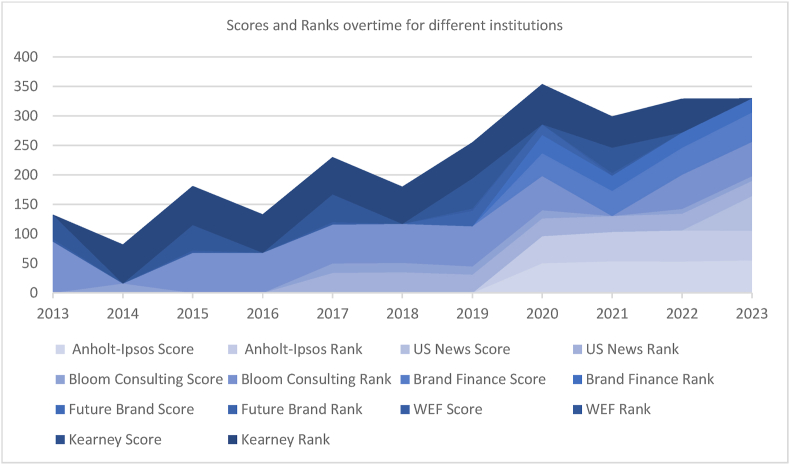
Temporal trends in scores and ranks.

Analyzing these scores and ranks over times reveals dynamic patterns and comparative performance, the graph below Fig. 3, shows how scores and ranks have changed over time, providing a complete picture. showcasing Qatar's significant progress over the past decade. Enhancements in tourism attractiveness, competitiveness, soft power and urban recognition demonstrate Qatar's efforts in nurturing its tourism sector and solidifying its position on the international stage.

Over the past decade, Qatar has undergone remarkable progress, evident across various indices measuring tourism attractiveness, competitiveness, soft power, and urban recognition. Notably, Qatar has experienced a notable surge in its rankings in key assessments such as the Country Brand Ranking Tourism by Bloom Consulting and The Travel & Tourism Competitiveness Index by World Economic Forum. These improvements signal a heightened perception of Qatar as an appealing tourist destination and a more competitive entity in the travel industry. Concurrently, Qatar's ascent in the Global Soft Power Index by Brand Finance underscores its increasing influence and global reputation. The overarching trend reflects an enhancement in Qatar's destination perception, coupled with the growing stature of its cities as global hubs. These developments are indicative of Qatar's sustained efforts in nurturing its tourism sector, bolstering its global image, and solidifying its position as a significant player on the international stage.

#### 4.3.2 Inbound tourism

Reflecting on Qatar's flourishing tourism landscape, a sport fan visitor among the participants who traveled to Qatar for the FWC exclaimed "*the dunes and the skyline (…) the mix of tradition and modernity is captivating (…) I'm definitely coming back to explore Qatar properly.*" This sentiment encapsulates the allure Qatar holds for travelers beyond mega-events. Qatar's inbound tourism industry is resilient and expanding due to continuous stream of international events and strategic tourism efforts. Saudi Arabia's focus on leisure tourism destination development has intensified competition for inbound tourists, alongside established locations in the UAE. Tourism in Qatar excels in safety, health, hygiene, business environment, and human resources. While it boasts a strong tourism and accessibility framework, its natural and cultural resources are less competitive, lacking tourist destinations that differentiate it from neighboring countries. Giving these insights, some researchers speculated that the FWC in Qatar may witness a lower supply of additional tourism. However, the WTTC's projections for 2022, the FWC year, meets the reality with 4 million tourists, and the numbers may continue rising to 7.4 million by 2030, reflecting a 20 % annual growth rate [48]. Official sources predict a 347 % rise in international visitors in 2023 over 2022 [25].

Guided by the Qatar National Tourism Sector Strategy 2030, Qatar Tourism is working with public bodies, policy advocates, tourism-enabling entities, private enterprises, and media to enhance the business environment, diversify the tourism portfolio, and boost visitor traffic and spending [25]. As emphasized by the industry expert interviewed, "*Qatar has done a remarkable job branding itself as a premium destination. The challenge now is widening its appeal*." Leveraging the legacy of the FWC, Qatar aims to expedite its tourism objectives and drive economic diversification, viewing it as "*an opportunity to accelerate [its] tourism goals and diversify the economy*," as stated by the participant from Qatar's tourism sector during the interview. Following the expansion of Doha Port, it has become a port of call for international cruises, presenting potential for increased inbound tourism. In 2018, a global marketing campaign with Qatar Airways encouraged travelers to have a free one-night stopover, with additional nights available for $50 per night. This initiative followed visa-free entrance for over 100 nationalities. Qatar has also established a robust offline presence in source countries to drive inbound arrivals.

As a result, Doha emerged as one of the most sought-after foreign destinations in 2021, in 2022, ranked first internationally by Skyscanner in 2022, and listed among Ctrip's top dream destinations for 2023 [34]. Moreover, international tourists spent 21 % more in Doha in 2022 than in 2019 [48]. By 2032, Doha is projected to rank 6th globally with $43 billion USD spent [48]. As underscored by the tourism industry insider interviewed, "*The World Cup put a global spotlight on Qatar – this has huge long-term potential for our industry*," highlighting the significant impact. Building on this momentum, the Middle East, including Qatar, experienced a remarkable resurgence. From January to July 2023, the Middle East, including Qatar, surpassed pre-pandemic levels by 20 % [49]. It stands as the only area to exceed 2019 levels, with Qatar experiencing a 95 % rise in arrivals [49].

## 5 Discussion

Discussing Qatar's destination image requires an exploration of its multifaceted nature, encompassing organic, induced, and modified induced images [4]. This understanding is crucial for a comprehensive evaluation of how the destination is perceived overall.

### 5.1 Universal pathway factors

Qatar's identity is molded by the pre- and during-visitation stages, akin to PCM theory's awareness stage [19]. These phases are intertwined with media, word-of-mouth, and promotional efforts, shaping both the organic and induced images of the destination. The cognitive dimension, rooted in individual beliefs and knowledge, constructs a mental image of Qatar as a destination. Meanwhile, the conative dimension reflects pre-visit goals or motives, aligning with push variables influencing travel decisions.

Strategic infrastructure improvement play a pivotal role in attracting tourists post-event, demonstrating the destination's commitment to preserving its allure beyond the event itself. It is noteworthy that Qatar's tourism sector has little influence on GDP prior to the event bidding phase, a pattern also observed in Russia hosting of the same event in 2018. This economic aspect underscores these nations' distinct relationships with tourism, and their ambitions to leverage mega-events for soft power [50].

As previously discussed, destination image is significantly influenced by worldwide media's value-laden and often unfavorable representation [51,29]. This is evident in how foreign media portray host countries, such as the negative coverage of South Africa [52] Russia's LGBTQIA + rights issues in 2018 [53] and Brazil's treatment of migrant workers in 2014 [54]. China's occupation of Tibet, treatment of minorities like the Uyghur, pollution, human rights, and democracy were also criticized during the Beijing 2008 Olympics [55]. Such media coverage can lead to a loss in destination image for host nations due to unfavorable publicity and diminished interest. For example, poor British news coverage resulted in reduced attendance forecasts for the South Africa World Cup.

### 5.2 Contextual factors

Contextual influences emerge during visitation, reflected in PCM theory's attraction and attachment stages [19]. In this phase, the modified induced image from personal destination experiences shapes visitors' perceptions. The affective dimension, involving real-life feelings, corresponds with external pull factors influencing travelers [17,18]. These destination-related factors enriches the overall travel experience, highlighting the complex interplay of various factors on destination image.

Safety, ethnic disturbances, socio-economic challenges, and infrastructure during the FWC in South Africa were highlighted worldwide, contributing to perceptions of risk and instability. This, along with Brazil's context, makes it challenging to determine if hosting the FWC improved the host country's image [54]. The concept of the *‘environmental bubble,’ as defined by* Horn and Breetzke [38, p.21] underscores the delicate balance between security and authenticity in tourism locations.

### 5.3 Outcomes

Qatar's inbound tourist business experienced significant growth post FWC 2022, showcasing resilience and development. Qatar hosts frequently sporting events (Doha 2023 FINA World Championships; 21st Asian Games 2024, 2030, 18th AFC Asian Cup 2023, Expo 2023 Doha), providing a continuous stream of service opportunities and display its hospitality and infrastructure. The 2022 FWC fostered location loyalty, emotional bonds, and repeat visits, akin to Germany's approach during the FWC 2006, to portray itself as a lovely and welcoming place to visit, countering worldwide perceptions about the Second World War and Nazism. In contrast, other hosting experiences, such as South Africa's (FWC 2010), Britain's (London 2012 Olympic and Paralympic Games), Brazil's (FWC 2014), and Russia's (FWC 2018) experiences, destination image did not improve following those mega-events. Various challenges are related, such as Brazil's and South Africa's lack of country branding, Britain's already strong international image and Russia's and challenges in managing media narratives during and after the event.

Qatar's successful hosting of the FWC has enhanced its image, making it a more appealing destination with lasting positive impacts. Notably, Qatar has seen improvements in familiarity, reputation, and cultural influence. This challenges the idea of a universal Occidental worldview and highlights the potential of sports to bridge cultural divides [38].

In response to challenges like high heat, alcohol restrictions, and unfavorable media coverage, Qatar's proactive measures demonstrate resilience and strategic planning for sustained growth. By effectively managing its destination image, Qatar has not only safeguarded its destination reputation but also set an example for future event hosts seeking positive and lasting impacts. The FWC has played a significant role in diversifying Qatar's natural and cultural resources, attracting visitors interested in unique cultural experiences, sporting events, and urban attractions. With continued innovation and differentiation in its tourism offerings, Qatar has the potential to emerge as a leading tourism destination in the Middle East and globally despite competition from neighboring countries investing heavily in leisure tourism.

To further enhance its position, Qatar must integrate sustainable practices into its tourism development to address environmental concerns and manage resources responsibly. The event's emphasis on environmental sustainability has positively influenced Qatar's destination image. Initiatives such as adopting sustainable building methods and energy-efficient technology reflects Qatar's commitment to environmental reforms. Additionally, the conversion of FWC stadiums into multi-sport arenas, and the donation of facilities to emerging countries for sports promotion underscore Qatar's dedication to long-term sustainability and global goodwill. Moreover, the compact nature of the FWC, with host cities conveniently located within a short distance from Doha [22], has contributed to significant environmental and cost savings.

## 6 Conclusion

This study makes a significant contribution to the existing literature by empirically validating previous assertions regarding the impact of mega-events on destination image, as evidenced by the case of Qatar. By examining how smaller nations strategically leverage mega-events to enhance their soft power and global appeal, this research underscores the transformative potential of such events on a nation's international perception. The nuanced exploration of Qatar's experience reveals the multifaceted nature of hosting mega-events, transcending conventional expectations and highlighting the need for a comprehensive understanding of destination image dynamics in the context of mega-events. Drawing on a variety of data sources, this study endeavors to enrich scholarly discourse and contribute to theoretical and practical insights in the realm of destination image and inbound tourism.

The findings underscore the need for a comprehensive approach to destination management, especially in the context of mega-events, to unlock their full socio-economic potential and enduring legacy. Policymakers and marketers can glean actionable insights from this study to design nuanced strategies and communication approaches that resonate with diverse stakeholders and capitalize on the lasting impact of mega-events on destination development. Moreover, this research contributes significantly to theoretical frameworks by enriching our understanding of the underlying mechanisms driving the influence of mega-events on destination image and tourism. It adds depth to existing theories by empirically validating their applicability in real-world scenarios and elucidating novel insights into the complex dynamics at play.

In terms of managerial implications, the study offers practical solutions for industry practitioners, moving beyond abstract principles to provide actionable recommendations grounded in empirical evidence. For instance, it highlights the importance of proactive destination branding initiatives, targeted promotional campaigns, and stakeholder collaboration to maximize the benefits of mega-events. Moreover, it emphasizes the need for long-term planning and strategic investments in infrastructure and hospitality services to ensure a lasting legacy and sustainable tourism growth. By translating research findings into tangible strategies and actionable steps, this study equips managers and practitioners with the tools to navigate the complexities of mega-event management effectively and capitalize on the opportunities they present.

While this study sheds light on critical aspects of destination image management, its scope is inherently constrained by the unique characteristics of nations like Qatar, characterized by limited natural attractions and challenging climatic conditions. Consequently, caution must be exercised in extrapolating the findings to nations with divergent contextual factors, as the applicability of the insights may vary. A nuanced understanding of these limitations is essential for a comprehensive evaluation of the study's findings and their implications for broader theoretical frameworks and practical applications.

Future research endeavors could enrich the scholarly discourse by employing confirmatory analyses to explore the enduring impact of mega-events on non-sport visitors' perceptions and loyalty, thereby enhancing our understanding of the sustained benefits of mega-events beyond the immediate event horizon. Additionally, comparative studies examining mega-event dynamics across diverse geographical and cultural contexts hold the potential to foster a more nuanced and globally relevant understanding of the intricate interplay between mega-events and destination development strategies. By embracing a transdisciplinary approach and leveraging emerging methodologies, future research can further elucidate the complexities of mega-event dynamics and inform evidence-based policy interventions aimed at maximizing the socio-economic dividends of hosting mega-events.

## Data availability

The data associated with this study has not been deposited into a publicly available repository but is available upon request. Please contact the corresponding author for access to the data.

## Additional information

No additional information is available for this paper.

## CRediT authorship contribution statement

**Mouna Hajjaj:** Writing – original draft, Validation, Resources, Project administration, Methodology, Investigation, Formal analysis, Data curation, Conceptualization, Software, Supervision, Visualization, Writing – review & editing. **Viktor Borodin:** Software. **Diana Claudia Perțicas:** Writing – review & editing. **Adrian Gheorghe Florea:** Funding acquisition.

## Declaration of competing interest

The authors declare that they have no known competing financial interests or personal relationships that could have appeared to influence the work reported in this paper.
